# Altered diversity and composition of gut microbiota in Wilson's disease

**DOI:** 10.1038/s41598-020-78988-7

**Published:** 2020-12-11

**Authors:** Xiangsheng Cai, Lin Deng, Xiaogui Ma, Yusheng Guo, Zhiting Feng, Minqi Liu, Yubin Guan, Yanting Huang, Jianxin Deng, Hongwei Li, Hong Sang, Fang Liu, Xiaorong Yang

**Affiliations:** 1 Clinical Laboratory, The First Affiliated Hospital of Guangdong Pharmaceutical University, Guangzhou, 510080 People’s Republic of China; 2 Department of Medical Laboratory, Guangdong Pharmaceutical University, Guangzhou, 510080 People’s Republic of China; 3 Center for Medical Experiments, University of Chinese Academy of Science-Shenzhen Hospital, Shenzhen, 518106 People’s Republic of China; 4 Department of Dermatology, Affiliated Hangzhou First People’s Hospital, Zhejiang University School of Medicine, Hangzhou, 310000 People’s Republic of China; 5 Department of Pathology, The First Affiliated Hospital of Guangdong Pharmaceutical University, Guangzhou, 510080 People’s Republic of China; 6 Department of Endocrinology, Shenzhen Second People’s Hospital, Health Science Center of Shenzhen University, Shenzhen, 518035 People’s Republic of China; 7 Institute of Biotherapy, Southern Medical University, Guangzhou, 510515 People’s Republic of China; 8 Nanjing School of Clinical Medicine, Southern Medical University, Jinling Hospital, Nanjing, 210002 People’s Republic of China

**Keywords:** Microbiology, Endocrinology

## Abstract

Wilson’s disease (WD) is an autosomal recessive inherited disorder of chronic copper toxicosis with high mortality and disability. Recent evidence suggests a correlation between dysbiosis in gut microbiome and multiple diseases such as genetic and metabolic disease. However, the impact of intestinal microbiota polymorphism in WD have not been fully elaborated and need to be explore for seeking some microbiota benefit for WD patients. In this study, the 16S rRNA sequencing was performed on fecal samples from 14 patients with WD and was compared to the results from 16 healthy individuals. The diversity and composition of the gut microbiome in the WD group were significantly lower than those in healthy individuals. The WD group presented unique richness of *Gemellaceae*, *Pseudomonadaceae* and *Spirochaetaceae* at family level, which were hardly detected in healthy controls. The WD group had a markedly lower abundance of *Actinobacteria*, *Firmicutes* and *Verrucomicrobia*, and a higher abundance of *Bacteroidet*es, *Proteobacteria*, *Cyanobacteria* and *Fusobacteria* than that in healthy individuals. The *Firmicutes* to *Bacteroidetes* ratio in the WD group was significantly lower than that of healthy control. In addition, the functional profile of the gut microbiome from WD patients showed a lower abundance of bacterial groups involved in the host immune and metabolism associated systems pathways such as transcription factors and ABC-type transporters, compared to healthy individuals. These results implied dysbiosis of gut microbiota may be influenced by the host metabolic disorders of WD, which may provide a new understanding of the pathogenesis and new possible therapeutic targets for WD.

## Introduction

Wilson’s (WD), also known as hepatolenticular degeneration, is a hereditary disorder characterized by accumulation of copper (Cu^2+^) in multiple vital organs such as brain, liver, etc*.* with an estimated prevalence of approximately 0.03–0.02‰ in the USA, Europe and Asia^1^. WD is also a genetic disease with complex phenotypes regulated by molecular genetics and epigenetic mechanisms^2^. The typically pathogenic mutations are in the copper-transporting gene, ATP7B, resulting in excess copper excretion through the biliary tract^2^. Recently published studies indicated that the gene expression in WD can be potentially modified by environmental and dietary factors. For example, regulation of the gene expression involved in methionine metabolism and epigenetic mechanisms could be affected by dietary factors, such as iron and methyl group donors^3^. Additionally, Penicillamine, a metabolic by-product of penicillin originated from the microbiota that avidly chelates copper, was the first choice for the treatment of WD^4^. We speculated that the intestinal microecology may contribute to the success action of Penicillamine, which prompted our interest in the interaction between WD and gut microbiome.

The gastrointestinal (GI) tract is the primary site of copper entry into the body, while genetic risk of autoimmunity has been showed to be related to distinct changes in the human gut microbiome. The human microbiome is determined by thousands of environmental, genetic and clinical factors and stochastic ecological processes, resulting in vast individual variation and biogeographic differences^5^. The composition of human microbial colonization is heritable beginning in the postpartum period and developing in an incremental manner. Moreover, the gut microbiota can influent the human health by providing crucial benefits to the development of the immune system, prevention of infections, acquisition of nutrients, and functionality of the nervous system^6,7^. Considering the intricacies of the interaction between the gut microbiota and the human host, it is essential to explore correlation between composition of human gut microbiota and WD. Meanwhile, the potential bacterial biomarkers need to be explored to develop the possible novel treatment strategies. Therefore, 16 s rRNA sequencing analysis of the gut microbiome was performed to profile fecal samples in relation to WD.

## Results

### Taxonomic analysis of 16S rRNA V4 amplicon sequence data

**Figure. 1:**
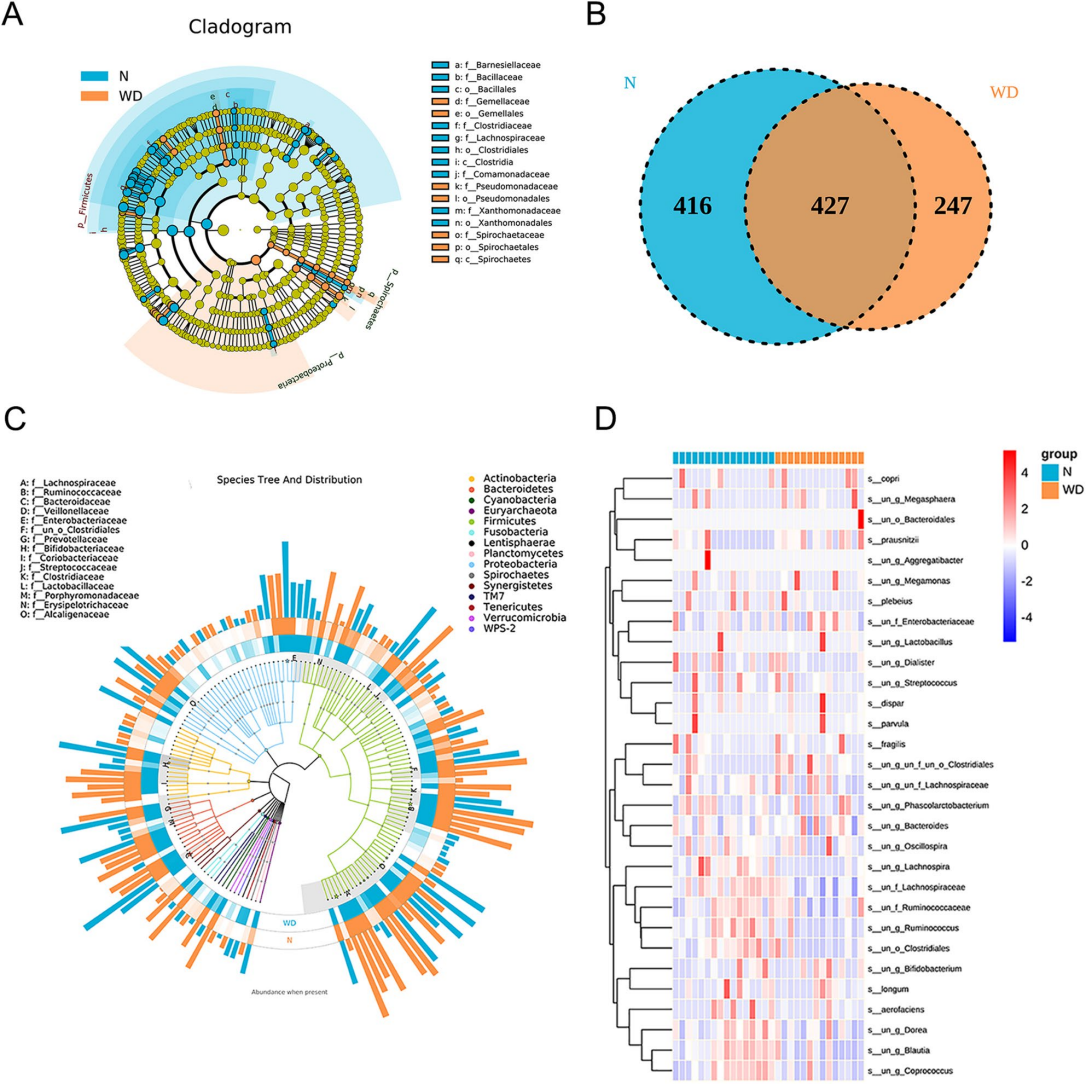
(**A**) LEfSe comparison of gut microbiota among WD and control groups, (**B**) Venn diagrams shared OTUs between different groups, (**C**) the species tree and distribution of gut microbial community, (**D**) the heatmap of gut microbiome among WD and control groups at species level.

The clinical index such as age, sex and body mass and BMI were comparable between two groups and summarized in Table1. To explore the characteristics of gut microbial community in WD patients, the microbiota relative taxon abundance was compared with that in healthy individuals. A total of 1604 operational taxonomic units were annotated for subsequent taxon-dependent analysis including 15 phyla, 85 families and 153 genera of gut microbes inferred from V4 amplicon sequencing with 97% similarity among the samples (Fig. 1A). The predominant genera were defined as one bacteria genus comprising greater than 1% of the total gut microbiota. The Venn diagram reflecting the difference between two groups as shown in Fig. 1B, exhibited 674 and 843 in WD and control group, respectively. The bacterial taxonomy distribution and heatmap of the WD group showed decreased density and clustering compared to healthy controls (Fig. 1C,D).

**Table1:** The baseline data of all participates.

Groups (*n*)	Gender (M/F)	Age (y)		Weight (kg)		Urinary copper excretion(mg/L)	
		Mean ± SD	*P* value	Mean ± SD	*P* value	Mean ± SD	*P* value
WD (n = 14)	9/5	32.2 ± 13.02	*t* = 1.042 *P* > 0.05	54.2 ± 10.33	*t* = 1.387 *P* > 0.05	726.44 ± 383.62	*t* = 6.966 *P* < 0.001
Control (n = 16)	9/7	37.8 ± 15.70		59.1 ± 9.05		58.65 ± 23.23

**Figure. 2:**
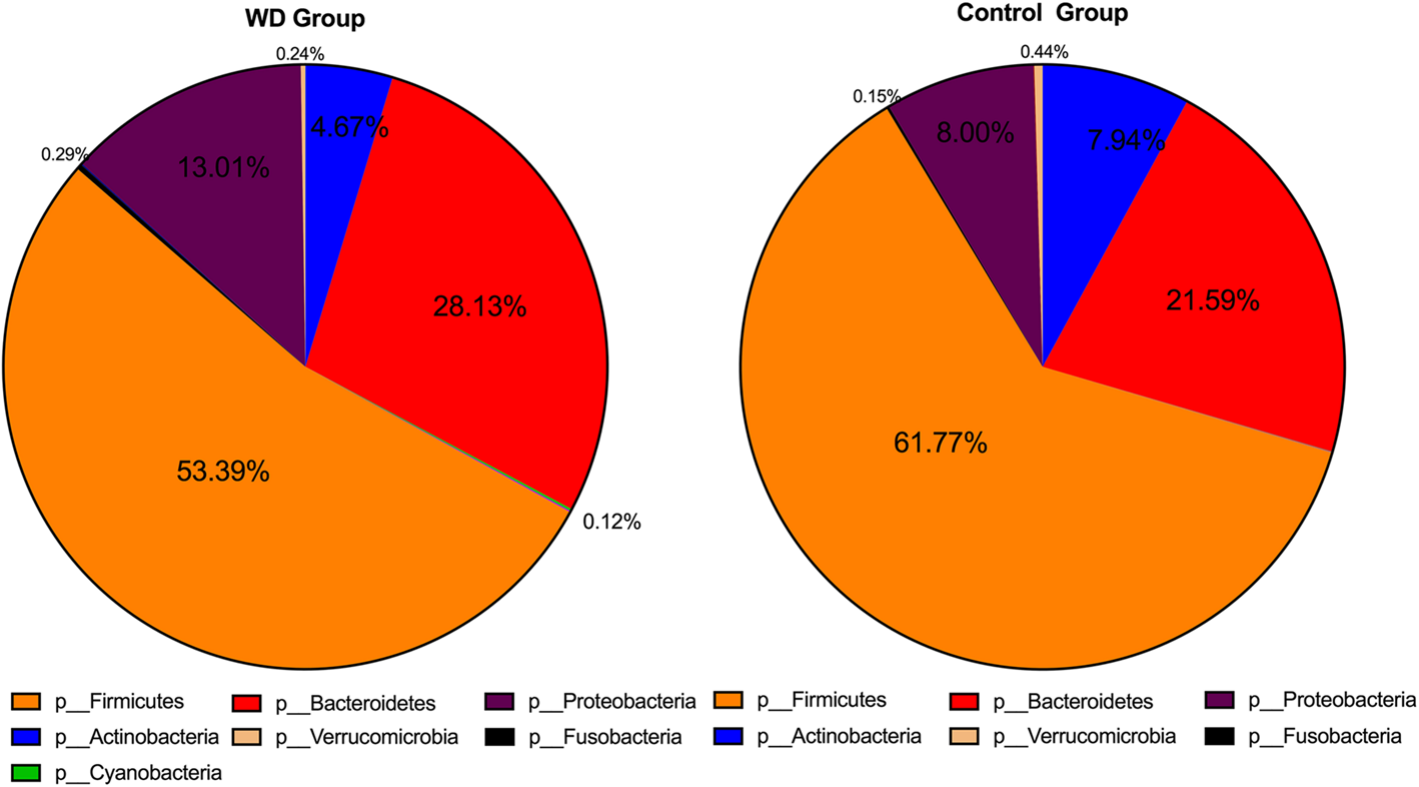
In the taxonomic profile, the OTUs were assigned to prevalent microbiome components of *Firmicute*s (53.39% vs. 61.77%, P < 0.05), *Bacteroidetes* (28.13% vs. 21.59%, *P* < 0.05), *Acidobacteria* (4.67% vs. 7.94%, P < 0.05), *Proteobacteria* (13.01% vs. 8.00%, *P* < 0.05), *Verrucomicrobia* (0.24% vs. 0.44%, P < 0.05) and *Fusobacteria* (0.29% vs. 0.15%, *P* < 0.05) at the phylum level.

At the phylum level, *Firmicutes*, *Bacteroidetes*, *Proteobacteria*, *Actinobacteria*, *Verrucomicrobia* and *Fusobacteria* were identified as six common phyla in both the WD group and control group, accounting for 99.73% and 99.89% of each whole gut bacteri (Fig. 2). The WD group had a markedly lower abundance of *Actinobacteria* (4.67% vs. 7.94%, *P* < 0.05), *Firmicutes* (53.39% vs. 61.77%, *P* < 0.05) and *Verrucomicrobia* (0.24% vs. 0.44%, *P* < 0.05), and a higher abundance of *Bacteroidetes* (28.13% vs. 21.59%, *P* < 0.05), *Proteobacteria* (13.01% vs. 8.00%, *P* < 0.05) and *Fusobacteria* (0.29% vs. 0.15%, *P* < 0.05), compared to healthy controls. The WD group also showed a higher abundance of *Cyanobacteria,* compared to that of healthy controls (0.12% vs. 0, *P* < 0.05) (Fig. 2). The ratio of *Firmicutes* to *Bacteroidetes* was decreased in the WD group, compared to healthy controls (1.90 vs. 2.86, *P* < 0.05).

**Figure. 3:**
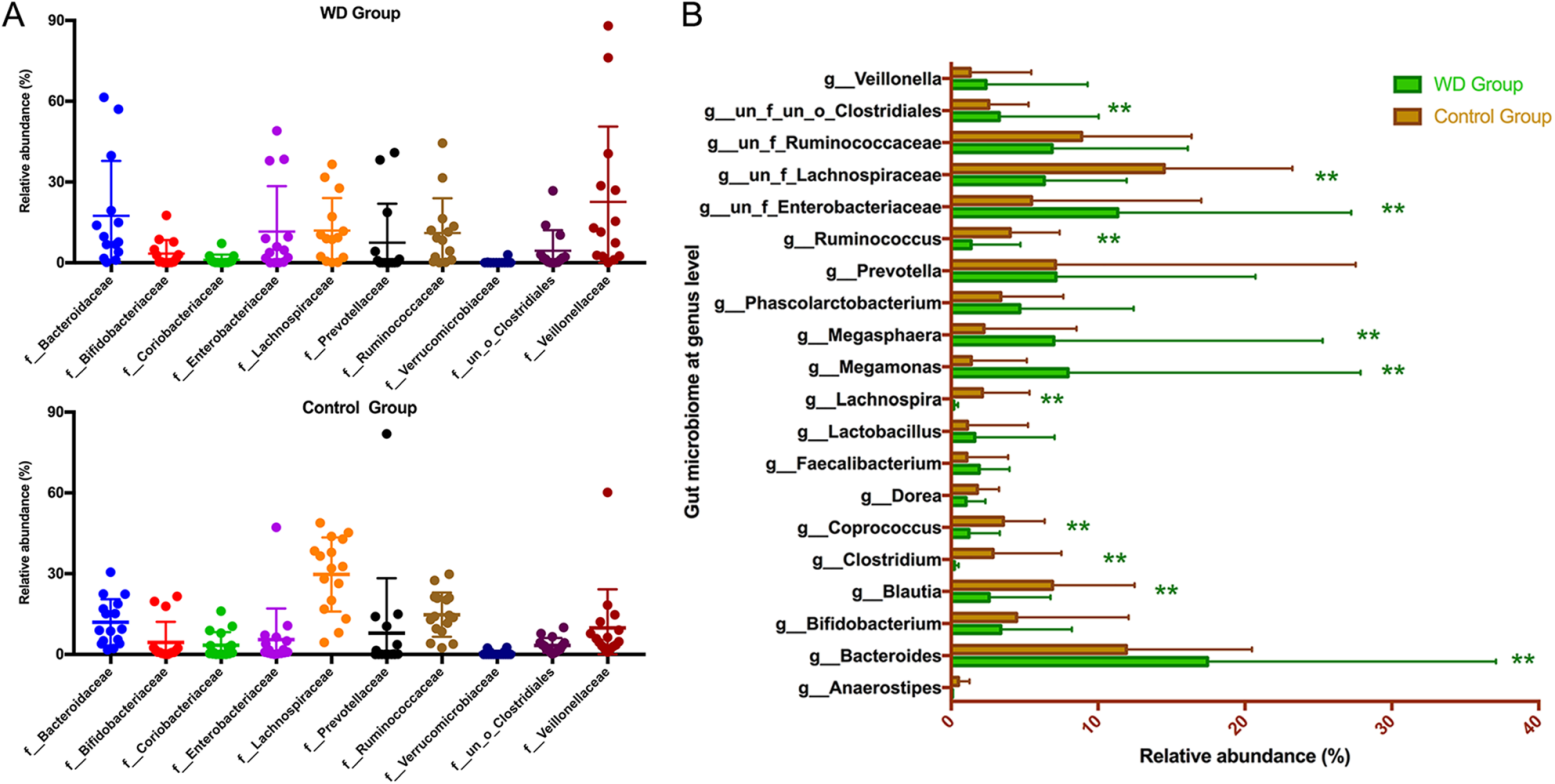
(**A**) Comparison of abundant components of the microbiome at the family level between the two groups. (**B**) Taxonomic summary of the gut microbiota of each group at the genus level. *Bacteroides*, *Enterobacteriaceae*, *Megamonas* and *Megasphaera* were significantly more abundant in the WD group than the control group (*P* < 0.05). *Lachnospiraceae*, *Ruminococcaceae*, *Blautia*, *Ruminococcus*, *Coprococcus Clostridium* and *Lachnospira* of WD group were significantly less abundant in the WD group than the control group (*P* < 0.05). Starred samples (*) showed significant differences between the two groups.

The internal individual variation in taxonomic structure was higher and more different dominant taxa were identified between two groups at the family level (Fig. 3A). Among the total families identified in the gut bacteria, 63 and 73 of the dominant families were respectively detected in the WD group and control group, respectively. *Lachnospiraceae* (phylum *Firmicutes*), *Ruminococcaceae* (phylum *Firmicutes*), *Bacteroidaceae* (phylum *Bacteroidetes*), *Veillonellaceae* (phylum *Firmicutes*), *Prevotellaceae* (phylum *Bacteroidetes*), *Enterobacteriaceae* (phylum *Proteobacteria*), *Bifidobacteriaceae* (phylum *Actinobacteria*) and *Coriobacteriaceae* (phylum *Actinobacteria*) represented eight most abundant components of the microbiome in WD group (Fig. 3A). While *Veillonellaceae*, *Bacteroidaceae*, *Lachnospiracea*e, *Enterobacteriaceae*, *Ruminococcaceae, Prevotellaceae*, *Clostridiales* (phylum *Firmicutes*) and *Bifidobacteriaceae* represented the eight most relative abundant components of the microbiome in control group.

The genus-level characterizations were more complex, with a number of bacterial genera showing significant differences between the two groups (Fig. 3B). In the WD group, *Bacteroides* (phylum *Bacteroidetes*), *Enterobacteriaceae* (phylum *Proteobacteria*), *Megamonas* (phylum *Firmicutes*), Prevotella (phylum *Bacteroidetes*), *Megasphaera* (phylum *Firmicutes*), *Ruminococcaceae* (phylum *Firmicutes*), *Lachnospiraceae* (phylum *Firmicutes*), *Phascolarctobacterium* (phylum *Firmicutes*), *Bifidobacterium* (phylum *Actinobacteria*), and *Clostridiales* (phylum *Firmicutes*) were the 10 most dominant components of 121 microbiota genera. While, the 10 most dominant components of the microbiota in 131 genera in the healthy control group were *Lachnospiraceae*, *Bacteroides*, *Ruminococcaceae*, *Prevotella*, *Blautia* (phylum *Firmicutes*), *Enterobacteriaceae*, *Bifidobacterium*, *Ruminococcus* (phylum *Firmicutes*), *Coprococcus* (phylum *Firmicutes*) and *Phascolarctobacterium.*

### Alpha diversity analysis of the gut microbiota

**Figure. 4:**
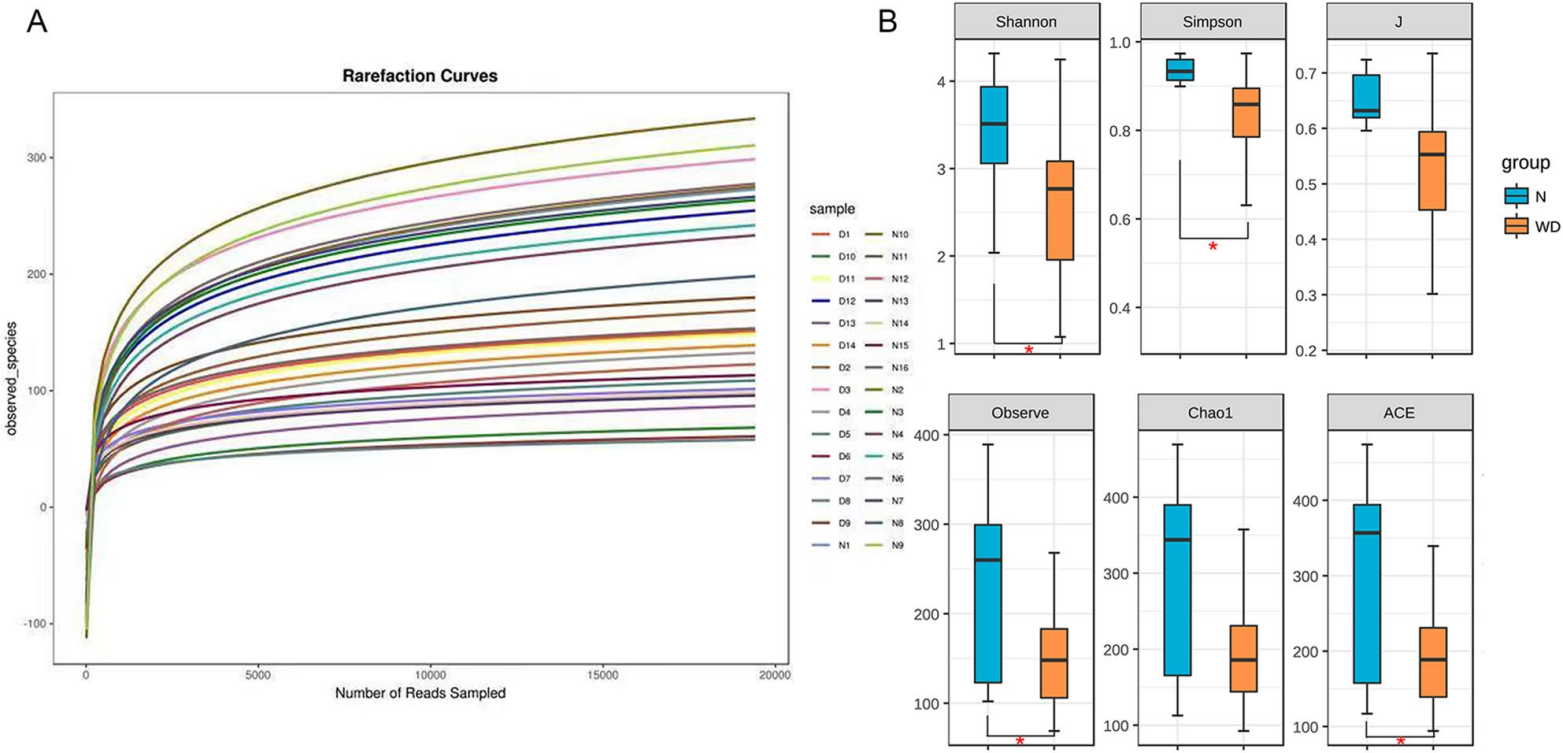
(**A**) The rarefaction curve was in a flat shape presenting reasonable quantity of the samples. (**B**) Comparison of the alpha diversity of the gut microbiota between the two groups, including species richness (represented by Chao1, Observed species (Observe), ACE and J) and evenness (represented by Shannon and Simpson index). The microbial community of the WD group had less diversity, richness and evenness than the control group. Starred samples (*) showed significant differences between the two groups.

To evaluate differences in the microbiota community structure between the two groups, the alpha diversities were analyzed as shown in Fig. 4. The rarefaction curve indicated the quality of samples is high and size is reasonable. The α diversity of the gut microbiota in the WD group was lower than that in the healthy control group in terms of the index of Shannon and Simpson (Shannon index − 28.509 *P* = 0.0369; Simpson index − 28.607, *P* = 0.0386). The results of Observe and ACE index were also statistically different between the WD group and control group (− 24.513, *P* = 0.0493 and − 24.902, *P* = 0.0357, respectively). There were no significant differences between the WD group and healthy control group in terms of richness index includes J and Chao1 (− 28.696, *P* = 0.061; − 21.848, *P* = 0.0784).

### Beta diversity analysis of the gut microbiota

**Figure. 5:**
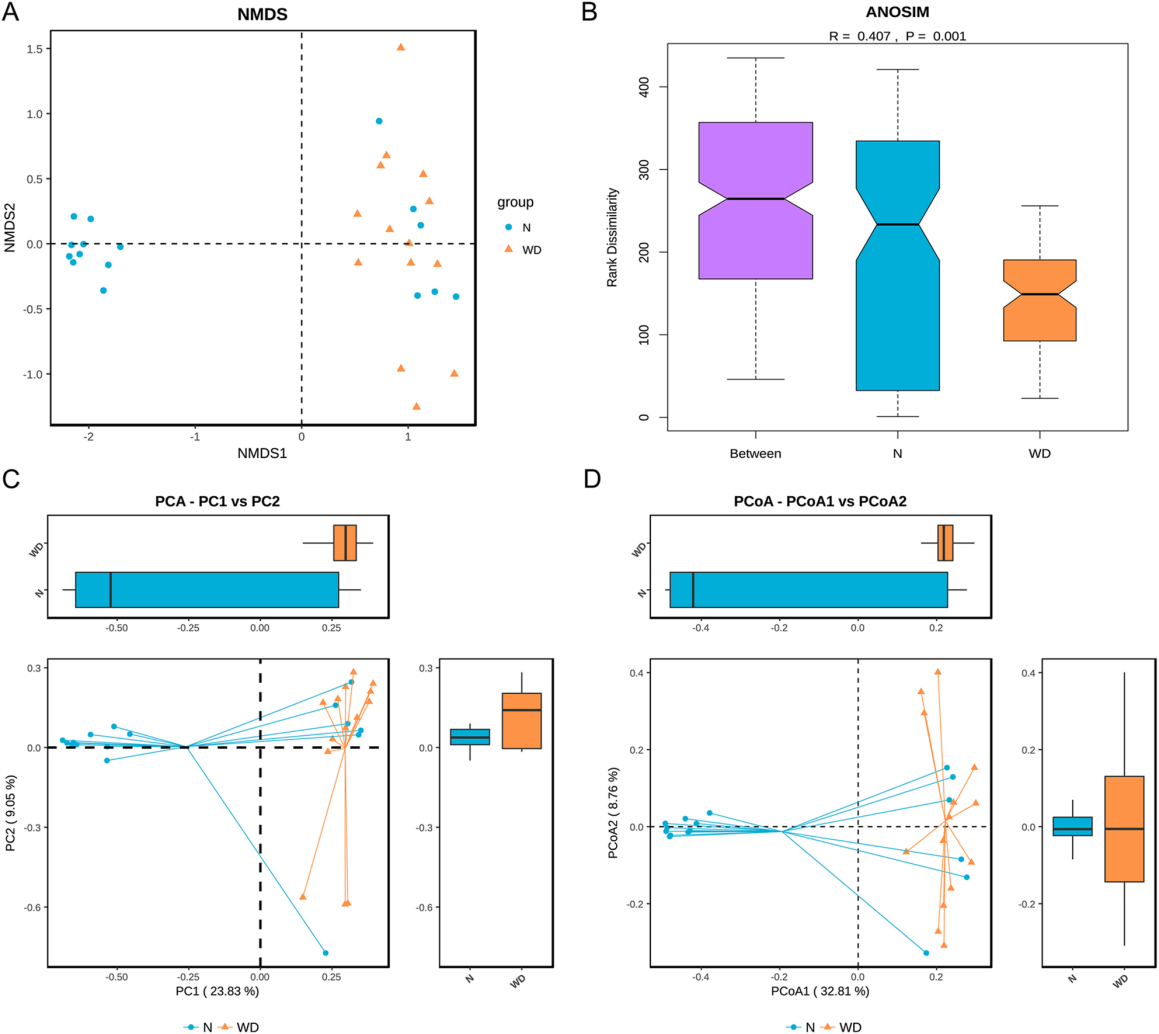
Comparison of the beta diversity of the gut microbiota between two groups. (**A**) Non-metric multi-dimensional scaling, (**B**) analysis of similarities (ANOSIM, R = 0.407, *P* = 0.001), (**C**) principal component analysis (PC1 23.83% vs. PC2 9.05%), and (**D**) principal coordinates analysis (PCoA1 32.81% vs. PCoA2 8.76%) illustrating the grouping patterns of the WD group and control group based on the Bray–Curtis and unweighted UniFrac distances. Each dot represents a sample, and the corresponding group can be found in the legend. Distances between any pair of samples represent the dissimilarities between each sample. There was a significant difference in β-diversity between the two groups.

Non-metric multi-dimensional scaling (NMDS) analysis revealed that the microbial genera were significant difference between WD patients and healthy controls based on the Bray–Curtis distances (Fig. 5A). The structure of the gut microbiota evaluated by ANOSIM was significantly different between the two groups (R = 0.407, *P* = 0.001) (Fig. 5B). Principal component analysis (PCA: PC1 23.83% vs. PC2 9.05%) and principal coordinates analysis (PCoA: PCoA1 32.81% vs. PCoA2 8.76%) based on unweighted UniFrac distance (Fig. 5C) and weighted UniFrac distance (Fig. 5D) indicated that the gut microbiome of WD patients clustered significantly separated from healthy controls.

### Functional profile of the gut microbiome

**Figure. 6:**
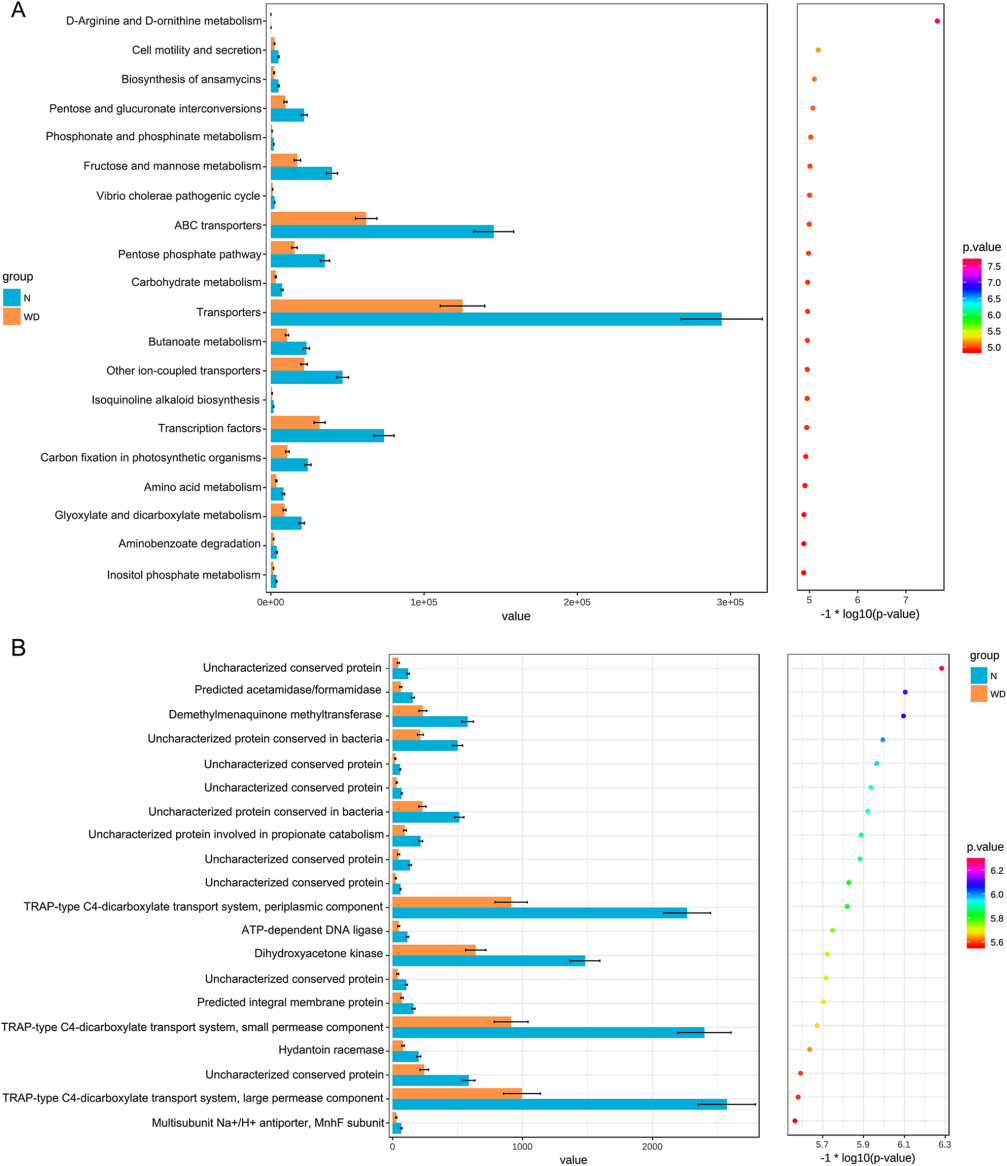
(**A**) Abundance in the KEGG pathway was lower in the WD group than in healthy controls (*P* < 0.001). (**B**) Abundance in the COG pathway was less in the WD group than in healthy controls (*P* < 0.001).

The functional composition of the gut microbiome was compared between WD patients and controls by KEGG and COG pathway analyses. Although the functional compositions of the two groups were highly similar, the gut microbiome in WD patients showed a less richness in pathways of transcription factors and ABC-type transporters than healthy controls (*F* = 31.745, *P* < 0.001). The analysis indicated the clustering of metabolic modules were decreased in WD group including metabolism of fructose (*F* = 31.2291, *P* < 0.001), mannose (*F* = 28.745, *P* < 0.001), butanoate (*F* = 31.385, *P* < 0.001), glyoxylate and dicarboxylate (*F* = 30.7384, *P* < 0.001), interconversions of pentose and glucuronate (*F* = 32.6781, *P* < 0.001), other ion-coupled transporters (*F* = 31.4571, *P* < 0.001), and carbon fixation in photosynthetic organisms (*F* = 30.6242, *P* < 0.001) (Fig. 6A). In addition, the TRAP-type C4-dicarboxylate transport system, periplasmic component (*F* = 38.397, *P* < 0.001), dihydroxyacetone kinas (*F* = 37.2417, *P* < 0.001), TRAP-type C4-dicarboxylate transport system, small permease component (*F* = 37.5488, *P* < 0.001), and TRAP-type C4-dicarboxylate transport system, large permease component (*F* = 36.4327, *P* < 0.001) were all significantly less abundant in the WD group than in the healthy controls. Similarly, demethylmenaquinone methyltransferase (*F* = 41.6979, *P* < 0.001) and uncharacterized protein conserved in bacteria (*F* = 41.9507, *P* < 0.001) and uncharacterized protein conserved in bacteria (*F* = 38.5353, *P* < 0.001) were also less abundant in WD patients than in healthy controls (*P* < 0.05) (Fig. 6B).

## Discussion

WD is an autosomal-recessive disorder characterized by abnormal copper metabolism leading to copper excretion disorder and deposition in target organs. Penicillamine is one of the standard therapies which was introduced as a treatment for WD by Walshe in 1956^8^. Penicillamine can combine with copper deposited in the tissue to form a soluble complex, and then excreted from the urine. Recently published study found that oral penicillin can induce gut microbiota dysbiosis in mice, which may associate with host lipid metabolism dysfunction and low-grade inflammation^9^. As all known, the human gut microbiome has been confirmed to play a causal role in the development of pathologies in animal models of metabolic disease such as obesity, Alzheimer’s disease and Type 1 diabetes (T1D)^10–12^. The human gut microbiome participates in nutrient metabolism, inhibition of pathogenic growth, angiogenesis stimulation, and the maturation and maintenance of the immunological system to ensure a balance or homeostasis in the host^6,13,14^. In light of the multiple roles of the gut microbiome played in a number of diseases and the satisfactory efficacy of Penicillamine on WD, it is necessary to explore the possible correlation between the gut microbiome and WD. Actually, the features of the gut microbiome in WD patients has been explored by Geng^15^. The results of two studies both indicated the disruption of the equilibrium in the gut microbiota of WD patients, exhibiting elimination, decreased density and loss of bacterial diversity in the microbial ecosystem. However, Geng only analyzed the features of gut microbiome in WD patients at phylum level. Though α and β diversity between WD and healthy controls were compared, the variation in details of different levels were lacked. The present study revealed some special and detailed characterizations of the gut microbiome in WD.

At the phylum level, the gut microbiome of the WD group showed a significantly lower abundance of *Firmicutes* compared to the healthy controls, covering the majority of butyrate-producing bacteria in human bacterial communities. However, the abundance of *Firmicutes* in WD patients reported by Geng was significantly higher than that of healthy controls (26.18% vs. 19.83%, respectively, *P* < 0.05)^15^. *Firmicutes* is known for its function in transforming undigested proteins and carbohydrates into acetic acid, and producing energy for the organism of host. The crucial short-chain fatty acids (SCFAs) butyrate participates in activating multiple physiological signal pathways, such as anti-inflammatory activities, and the differentiation and proliferation of regulatory T cells^16,17^. We speculated that the alteration of bacterial composition may cause a low concentration of intestinal SCFAs, leading to skewed physiological functions in WD patients. Subsequent KEGG and COG pathway analysis further confirmed reduced transport and metabolism functional groups in the gut microbiome of the WD group, which intensify the correlation between WD and the gut microbiome. We thus speculate that the WD triggers dysfunction of gut microbiota and increases intestinal permeability, interfering with the intestinal microbiota by transplanting fecal bacteria rich in SCFAs, and supplementing with butyric acid may be effective therapies for WD.

Meanwhile, the WD group showed significantly decreased phylum levels of *Actinobacteria* and *Verrucomicrobia* compared to the control group. The *Actinobacteria* is known to have the capability of surviving in extreme, even toxic environments^18^. *Verrucomicrobia* is mucin-degrading bacteria residing in the intestinal mucosa that contribute to intestinal health and glucose homeostasis, and plays as an interface between the human gut microbiome and host tissues^19^. Therefore, the decrease of these two probiotics may cause the disorder of physiological functions in WD patients. The gut microbiome in the WD group also showed a significantly higher abundance of *Proteobacteria* and *Fusobacteria* than in healthy individuals, consistent with the results reported by Geng^15^. The opportunistic pathogen of *Proteobacteria* created a major structural imbalance of gut microbiota in WD patients, while *Fusobacteria* have been widely recognized for the potential inducer of T regulatory cells or carcinogens promoting autophagic activation^20^. The WD group also showed higher abundance of *Cyanobacteria,* which has ability to perform nitrogen and carbon fixation and are involved in complex metabolic pathways with different mechanisms. Moreover, *Cyanobacteria* have an exceptionally high iron demand because of their involvement in the functions of a variety of crucial enzymes^21^. We speculated that microbiota-derived products may be act as triggers for promoting a proinflammatory and metabolically dysfunctional environment in WD patients. Microbiota-produced butyrate has been reported to provide energy for colonocytes and then prevent autophagy in the intestine of host^17^. Additionally, physiological homeostasis may be disrupted by gut microbiota, resulting in host metabolism disruption, and immune, neurological or cognitive system dysregulation and others^22,23^. Therefore, the decreased abundance of ABC transporters system signaling pathways further confirmed potential alteration of energy metabolism related to gut microbiota in WD. To our best knowledge, it is the first report to analyze the correlation between transporters system signaling pathways and gut microbiome of WD.

We also noticed that WD patients exhibited a lower ratio of *Firmicutes* to *Bacteroidetes* than healthy individuals. The dysbiosis of gastrointestinal tract metabolism was reported to be associated with low *Firmicutes*/*Bacteroidetes* ratio, causing a low concentration of circulating SCFAs, influencing elements of immune and inflammation system. These observations suggest that the gut microbiome has a potential impact on conserved functions, which may further inducing the pathogenesis of WD. Human lymphocyte antigen (HLA) gene alleles have been confirmed to have a significant effect on the composition of the gut microbiota in late infancy^18^. We thus speculated that some metabolic product of gut bacteria may be recognized by high risk genotypes in WD leading to alteration of the taxa in the human gut.

The features of gut microbiome in WD group at the family and genus levels were more complex and varied significantly from the control group, presenting a different pathogen microbiome and well characterized structures. The *Bacteroides* showed a greater abundance in the WD group than in healthy individuals. *Bacteroides* are correlated with diets high in animal protein and saturated fats. Moreover, the *Bacteroides* genus was reported to enhance the efficacy of the anti-CTLA4 immune checkpoint in mice and is speculated to contact and stimulate T cells and DCs directly by means of pathogen-associated molecular patterns in the host^24^. The *Enterobacteriaceae* were markedly more abundant in WD than control group. The significant opportunistic pathogen *Enterobacteriaceae* is present in the human gut without causing symptoms or diseases under normal circumstance. However, host immunity and environmental factors, such as redox state and availability of oxygen, may result in significant variation of *Enterobacteriaceae*. The richness of *Enterobacteriaceae* in WD further suggested the correlation between pathogen microbiome and WD. The development of targeting identified bacterial biomarkers of gut microbiome may be an alternative therapeutic strategy to WD in future. The gut microbiome of controls and WD patients has a comparable abundance of *Prevotella* genus, which is more universal in populations with high carbohydrate and sugar consumption, as observed in agrarian and vegetarian societies^25^. Furthermore, the WD group exhibited significantly lower abundance of *Blautia*, *Ruminococcus* and *Coprococcus* genus in comparison with control group, which are considered as important components of commensal microbiota and play important roles in several homeostatic functions such as immunity, neurohormones and metabolism. *Blautia* plays a role in digestion of complex carbohydrates and also shows decreased abundance in diabetes, irritable bowel syndrome, Crohn’s disease and and nonalcoholic fatty liver diseases^26^. *Coprococcus* is a genus of anaerobic cocci which is part of the human faecal microbiota that functions in producing butyrate^27^. The decreased of these probiotics indicated the defections of multiple physiological function presented in WD may be caused by the dysbiosis of gut microbiome. Additionally, *Megamonas*, *Megasphaera* and *Clostridiales* are genera of *Firmicutes* bacteria that showed increased abundance in the WD group. *Clostridiales* includes bacterial species that produce short-chain fatty acid, which are important for the equilibrium between regulatory T cells and helper T type17 (Th17) cells^23^. These results further indicated the structure of bacterial communities is more various than previous study and some potential gut microbiome may be identified as bacterial biomarkers. In addition, the analysis of KEGG and COG pathway indicated that these bacteria affect the host by shedding different microbial bioactive molecules, including transcription factors, metabolism of fructose, mannose, butanoate, glyoxylate and dicarboxylate, different transporters system such as ion-coupled transporters, ABC tranporters and TRAP-type C4-dicarboxylate transport system. Increasing evidences demonstrated that gut microbiota related transporters system appears to be key microbial bioactive signaling pathways. These transport systems regulate the energy system by promoting utilization of glucose, ribose/galactoside. We speculated that the dysfunction of gut microbiota in WD may influent the metabolic of copper via transporters system signaling pathways. In the next step, specific metabolites of the microbiome may be searched for as therapeutic chemicals like penicillamine in WD.

However, the main limitation of the study is the small samples size causing from rarity which may affect the accuracy of the results. Meanwhile, more advanced method including metagenomics and metabonomics need to be applied to explore the possible specific metabolic or microbiota for WD. To compensate for these weaknesses, cooperation among multi-medical center need to fully elucidate the intestinal flora and treatment of this disease.

In summary, this study analyzed the features and possible functions of gut microbiota in WD patients, providing a new understanding of the pathogenesis of WD and identified potential bacterial biomarkers. The structure of the microbiota in WD patients showed an elimination, low-density, microbial ecosystem with loss of bacterial diversity. Both COG and KEGG pathway analysis indicated decreased abundance of some dominant metabolism and transport related pathways in WD patients. The dysbiosis of gut microbiota may contribute to host metabolic disorders of WD. The results may provide new insights and will facilitated future studies of the pathogenesis of WD and the development of novel treatment strategies. Under these conditions, fecal transplant and microbiota-related metabolites may hold promise for the prevention of WD.

## Materials and methods

### Samples

Fecal samples were obtained from 14 newly diagnosed WD patients and 16 healthy participants for subsequently sequencing of 16S rRNA. The diagnosis of WD was confirmed by ATP7B gene and satisfy clinical diagnostic criteria including family history, clinical manifestations, neurological examination, low level of serum ceruloplasmin, high urinary copper excretion in 24 h, examination of liver function, ultrasonography of the liver, and magnetic resonance imaging (MRI) of the brain. Patients with any of the following conditions were excluded: respiratory or renal failure, congestive cardiac disease, severe liver dysfunction, or being prescribed with probiotics or antibiotics within 1 month before admission. The control group was consisted of 16 healthy participants who did not take any probiotics or antibiotics within 1 month before inclusion. The clinical characteristics of all subjects were summarized in Table 1 and the parameters such as age, gender and weight were comparable between two groups (*P* > 0.05). The fresh fecal samples from all participates were collected into sterile eppendorf tubes and were frozen at − 80 °C refrigerator immediately, until the extraction of DNA. Each patient signed written informed consent before inclusion in this study. All experiments were approved and carried out in accordance with the guidelines of the Ethics Committee of the First Affiliated Hospital of Guangdong Pharmaceutical University.

### DNA extraction, amplification, and sequencing of the V4 region of the bacterial 16S rRNA gene

Microbial DNA was extracted from 30 fecal samples using a PowerSoil DNA Stool Mini Kit (MoBio) according to the protocol recommended by manufacturer. Briefly, the V4 variable region of the bacterial 16S rRNA gene was used to amplify by polymerase chain reaction (PCR) with universal primers 806R (GGACTACHVGGGTWTCTAAT) and 341F (CCTACGGGNGGCWGCAG). The PCR conditions consisted of predenaturation step at 95 °C for 2 min followed by 30 cycles denaturation at 95 °C for 30 s, annealing at 52 °C for 30 s, and extension at 72 °C for 45 s, with a final extension step at 72 °C for 5 min. The PCR products were visualized by the method of agarose gels electrophoresis. Finally, equimolar amounts of purified amplicons were pooled and subjected to paired-end sequencing on an Illumina HiSeq/MiniSeq benchtop sequencer (Illumina) for genome analysis^28,29^.

### The analysis of Microbiome data

The raw FASTQ files were initially de-multiplexed, and then quality-filtered by Chimera check and merged using FLASH with the processed sequences according to the BIPES protocol.10 and Quantitative Insights Into Microbial Ecology (QIIME) 1.9^28,29^. Briefly, forward and reverse bacterial 16S rRNA reads were merged with a minimum merge length of 200 bp, singletons and chimeras were simultaneously removed by filtering in UPARSE. Operational taxonomic units (OTUs) were defined as a sequence similarity level of 97% and assigned to taxon-dependent analysis through the Ribosomal Database Project (RDP) classifier by the Green Genes Database to explore WD-associated differences in the human gut microbiota. Based on OTUs analysis, the index of Ace, observed species, Shannon, Chao1, Simpson and J were used to calculate alpha diversity metrics. Principal component analysis (PCA) and principal coordinate analysis (PCoA) were then analyzed to compare the microbial composition between the samples. The unweighted-pair group method with arithmetic mean (UPGMA) trees was created based on unweighted UniFrac distance matrices. The statistical significance of difference between groups was evaluated by analysis of similarities (ANOSIM). The databases of Kyoto Encyclopedia of Genes and Genomes (KEGG) and Cluster of Orthologous Groups (COG) were used to analyze the pathway richness by PICRUSt^30,31^. Finally, the influence of each differentially abundant gut microbiota was evaluated by the method of linear discriminant analysis (LDA) effect size (LEfSe).

### Statistical analysis

Statistical tests were performed using R 3.0.3 software (R Foundation for Statistical Computing) and Prism software (Graph Prism 7.0 Software Inc. CA, USA). The diversities between two groups were compared by Wilcoxon’s rank-sum test. The categorical variables were analyzed by Fisher’s exact test. The chi-square test was used for categorical variables. In all analyses, a value of *P* < 0.05 considered statistically significant difference in the compared groups.

### Conference presentation

This article is present on a university repository website and can be accessed on https://www.researchsquare.com/article/rs-15131/v1.

## Data Availability

All data used during the study are available from the corresponding author by request.
